# Deep Learning-Based Device-Free Localization Scheme for Simultaneous Estimation of Indoor Location and Posture Using FMCW Radars

**DOI:** 10.3390/s22124447

**Published:** 2022-06-12

**Authors:** Jeongpyo Lee, Kyungeun Park, Youngok Kim

**Affiliations:** Department of Electronic Engineering, Kwangwoon University, Seoul 01897, Korea; jp.lee.life@gmail.com (J.L.); ruddmsdl0651@naver.com (K.P.)

**Keywords:** indoor localization, posture estimation, deep learning, FMCW radar

## Abstract

Indoor device-free localization (DFL) systems are used in various Internet-of-Things applications based on human behavior recognition. However, the usage of camera-based intuitive DFL approaches is limited in dark environments and disaster situations. Moreover, camera-based DFL schemes exhibit certain privacy issues. Therefore, DFL schemes with radars are increasingly being investigated owing to their efficient functioning in dark environments and their ability to prevent privacy issues. This study proposes a deep learning-based DFL scheme for simultaneous estimation of indoor location and posture using 24-GHz frequency-modulated continuous-wave (FMCW) radars. The proposed scheme uses a parallel 1D convolutional neural network structure with a regression and a classification model for localization and posture estimation, respectively. The two-dimensional location information of the target is estimated for localization, and four different postures, namely standing, sitting, lying, and absence, are estimated simultaneously. We experimentally evaluated the proposed scheme and compared its performance with that of conventional schemes under identical conditions. The results indicate that the average localization error of the proposed scheme is 0.23 m, whereas that of the conventional scheme is approximately 0.65 m. The average posture estimation error of the proposed scheme is approximately 1.7%, whereas that of the conventional correlation, CSP, and SVM schemes are 54.8%, 42%, and 10%, respectively.

## 1. Introduction

Several indoor services based on human recognition, such as the presence, location, and postures, have been introduced for realizing the goals of the fourth industrial revolution. These services form the basis for various Internet-of-Things applications, sensing and controlling systems, and security operations [1]. Therefore, indoor localization schemes have been increasingly investigated in recent years. Based on the service scenarios, the two categories of indoor localization schemes include active and passive localization schemes [2]; the latter are also referred to as device-free localization (DFL) schemes. In the case of active localization schemes, the target requires electronic equipment, such as radio-frequency identification (RFID) tags, smartphones, and wearable devices, to interact with the anchor nodes in wireless sensor networks (WSNs) [3,4,5]. Conversely, the target does not require any electronic equipment in the case of DFL schemes.

In comparison with active localization schemes, DFL schemes exhibit unique superiority in various applications, such as security systems, safety monitoring systems, patient or elderly health monitoring systems, search-and-rescue operations, and posture recognition systems in hospitals or disasters, wherein the targets rarely carry electronic devices [6,7,8,9,10]. Wireless sensing schemes have been researched as promising DFL schemes for indoor applications [11,12,13]. As wireless sensing schemes exploit the effects of human existence on the received wireless signals over the WSN, several transmitters and receivers are required in a relatively small space, rendering the schemes less practical. Although several intuitive approaches using cameras have been introduced as competitive DFL schemes [6,7,14,15], their usage in dark environments and during disaster situations is limited, as it is difficult to provide clear visibility owing to the presence of smoke or mist. Moreover, certain privacy issues are associated with camera-based DFL schemes. Therefore, DFL schemes with radars [16,17,18] were considered, as they are known to prevent privacy issues and function efficiently even in dark or smoky environments.

As part of conventional indoor localization technology, multilateration and fingerprint methods are well-established techniques that use radio signals, such as Wi-Fi, Zigbee, RFID, Bluetooth, and frequency-modulated continuous-wave (FMCW) radars [19,20,21,22]. As the multilateration method is based on range estimation, its localization performance relies on the accuracy of the estimated distance to the target [23]. Conversely, the performance of the fingerprint method relies on the accuracy of the correlation between the pre-collected and real-time data; herein, the real-time data collection is considered a random process [24]. With the advent of the fourth industrial revolution, artificial intelligence (AI) technology has been widely applied in various fields, including computer vision and speech recognition [25]. Furthermore, AI technology is employed in the localization field and in conventional machine learning (ML) algorithms, such as common spatial patterns (CSP) [26], support vector machine (SVM) [27], and deep learning (DL) [28]. Therefore, AI is employed in DFL schemes, exploiting FMCW radars to enhance the accuracy of distance estimation [29]. Recently, a DL-based indoor two-dimensional (2D) localization scheme was introduced using a 24-GHz FMCW radar to address the limitations of the conventional 2D localization scheme based on multilateration methods [18].

Although several approaches have been introduced for the localization of device-free targets with radar, simultaneous estimation of location and posture has not been investigated sufficiently despite the requirement of both details of the target for various application scenarios. For instance, if both location and current posture of the target are known, a system can quickly respond to search-and-rescue operations when an accident occurs. In this study, we propose a DL-based DFL scheme for simultaneous estimation of indoor location and posture using 24-GHz FMCW radars. The proposed scheme estimates the 2D location information of the target for localization, and four different postures, namely standing, sitting, lying, and absence, are estimated simultaneously. The effectiveness of the proposed scheme was evaluated experimentally, and its performance was compared with that of conventional schemes under identical conditions.

The primary contributions of this study can be summarized as follows:

1. A DL-based scheme is proposed for simultaneous estimation of the indoor location and posture of a device-free target using 24-GHz FMCW radars. Unlike most existing studies that focused on the 2D localization of the target, we propose a simultaneous indoor location and posture estimation scheme based on the DL technique. A parallel convolutional neural network (CNN) structure with a regression model for localization and a classification model for posture estimation are included in the proposed scheme.
2. The developed simultaneous location and posture estimation system comprises two FMCW radars and a server to experimentally evaluate the performance of the proposed scheme. The developed system collects the time-series radar data at each location, and the collected data are divided into training and testing datasets to evaluate the proposed DL model.
3. The performance of the proposed scheme is compared with that of conventional schemes under identical experimental conditions. Particularly, the performance of posture estimation is analyzed using the proposed scheme and conventional CSP and correlation schemes in terms of both false-positive (FP) and false-negative (FN) errors to ensure an unbiased comparison.

The remainder of this paper is organized as follows. Section 2 briefly reviews the relevant literature and compares the merits and demerits of existing methods. In Section 3, the developed simultaneous location and posture estimation system is described, and conventional schemes are introduced. Section 4 presents the proposed simultaneous indoor location and posture estimation scheme based on the DL technique. The performance of the proposed scheme is evaluated through experiments, and the results are explained in Section 5. Finally, Section 6 summarizes the conclusions of this study.

## 2. Related Works

In this section, we summarize some related works about human posture or activity recognition and localization applications in indoor environments. In the first stage of research on localization, researchers investigated active schemes that require the target to carry a wearable device, such as a motion sensor or beacon [30,31,32,33,34,35]. For instance, Wang et al. [33] proposed a single person fall detection system, namely WiFall, which can be used to monitor elderly people with physical conditions when they stay at home alone.

However, the active scheme requires the cooperation of targets, who are likely to be disturbed by the presence of equipment that inconveniences them. Moreover, it is considered to be only a part of the localization technique, because it does not aid in localizing a device-free target.

To address these drawbacks of active schemes and extend the possible applications of localization systems, DFL schemes using cameras or radio signals, such as Wi-Fi, Zigbee, RFID, Bluetooth, and FMCW radars, were introduced [6,7,8,9,10,11,12,13,14,15,16,17,18,19,20,21,22]. Bhattacharya et al. [34] developed a fall detection and breathing sensing technique based on radar technique to detect a fall after it has happened even when the person is static. Sun et al. [35] proposed a RF sensing method to achieve joint moving target localization and activity or gesture recognition by a dual-frequency continuous wave radar.

As a promising technology for detecting and recognizing human activity, several intuitive approaches using cameras were introduced, which classify human motion into three major areas to recognize human activity using single-view or multi-view cameras [36]. Owing to the development of high-performance computer hardware and the remarkable performance of AI technology, recent camera-based DFL schemes have achieved improved performance by employing ML algorithms [37,38]. However, camera-based DFL schemes require extremely high computing power owing to the high resolution of images. Additionally, they are vulnerable to the infringement of personal information. The limited usage of camera-based DFL schemes in dark environments and during disasters is another disadvantage.

Wireless sensing schemes that exploit the characteristics of wireless signals, such as the received signal strength (RSS) or channel-state information, have been introduced as another promising DFL scheme. Wilson and Patwari introduced a radio tomography imaging (RTI) scheme, which used RSS variations to obtain the image of a device-free target and modeled the DFL as an RTI problem [39]. To reduce the computational resource requirements of the imaging-based localization method, Talampas and Low introduced a low-computational geometric filter algorithm to address the DFL problem [40]. However, the aforementioned methods generate each wireless node in the high-density WSN, which requires multiple transmitters and receivers to be placed in a relatively small space. Recently, Ninnemann et al. introduced a channel impulse response (CIR)-based method that locates a device-free target by analyzing the reflections of wireless signals from the target in a WSN [41]. However, their scheme requires the CIR to be collected from different transmission channels between multiple anchors for the localization of a target. Moreover, they did not sufficiently consider the severe multipath effects in indoor environments.

Radar-based DFL schemes are increasingly investigated as they can prevent privacy issues and function efficiently even in dark or smoky environments. Indoor DFL schemes using FMCW radars with various bandwidths have been introduced because their performance is closely associated with the frequency band. Typically, the hardware is calibrated to increase the accuracies of both target detection and localization with higher bandwidth. In the conventional 2D DFL scheme with FMCW radars, the distance to the target is initially estimated using a radar. Subsequently, multilateration methods are employed to estimate the 2D location of the target [18]. The state-of-the-art conventional scheme exhibits relatively low errors in 2D localization with an accurate estimated distance.

However, owing to the random nature of radio signals in indoor environments, distance estimation tends to be slightly inaccurate [42,43]. Therefore, a distance estimation scheme based on DL technology was introduced to enhance accuracy [29].

Radar-based device-free human activity or posture recognition systems have more convenience and practicality, which does not strongly depend on the electric equipment worn. Vaishnav et al. [44] present a novel fused human localization and activity classification using unscented Kalman filter and LSTM classifier, they demonstrated results by a short range 60-GHz FMCW radar. Abdu et al. [45] considered elderly fall detection issues, and they integrated the FMCW radar signal micro-Doppler features extracted from the CNN, using an SVM classifier to complete activity classification tasks. Both the authors in [46,47] exploited the features of the short-time Fourier transform (STFT) from 24 GHz FMCW radar signal to recognize the subject’s physical activity.

Compared with conventional indoor localization methods, DL-based indoor localization and human sensing techniques have been widely developed recently [48,49,50,51]. The advantage of DL over conventional methods is its representation ability, which is the capability to automatically discover the needed features [52]. Yin et al. [53] proposed a new localization algorithm to correct erroneous data through an anomaly detection algorithm based on a deep neural network (DNN). Most works focused on a 2D CNN-based model to process images or image-like data for localization [54,55]. Taking the time-series data into consideration, Zhang et al. [56] proposed an LSTM neural network positioning method by optimizing the channel state information (CSI) amplitude and phase data feature ratio. Kim and Moon introduced a deep CNN scheme for human detection and activity classification using the Doppler radar [57]; however, the 2D location of the target was not considered. Although DFL schemes with radars have been introduced for the localization of device-free targets, simultaneous estimation of location and posture are not sufficiently investigated despite the requirement of both details for various application scenarios. In the previous study using FMCW radar [18], DL-based localization was presented, but only two-dimensional location could be estimated. In this study, we propose a DL-based DFL scheme that can simultaneously acquire height information by estimating the posture of the target as well as the indoor 2D location. We have made it easy to implement the extension of functions through a combination of simple networks.

## 3. System Description

### 3.1. FMCW Radar Principle

**Figure 1:**
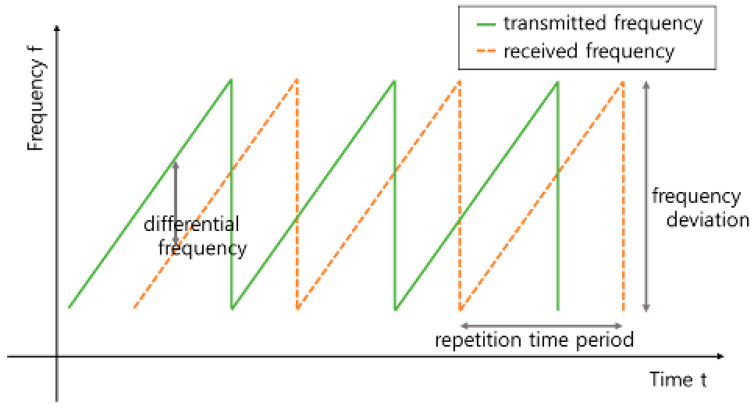
Time-dependent frequency shape of FMCW radar.

The FMCW radar emits a serrated signal waveform that changes frequency linearly over time (see Figure 1); subsequently, the radar receives the reflected signal from the target. As the distance between the radar and a target generates a propagation delay, the frequency of the received signal differs from that of the currently generated signal; the frequency is proportional to the delay time. Using this relationship, the delay time is obtained from the differential frequency, and the distance to the target can be estimated as follows:

$$distance=\frac{C*T*f_{d}}{2*\Delta f}\;,$$

where C is speed of light, T is repetition time period, $f_{d}$ is differential frequency, Δf is frequency deviation.

### 3.2. System Overview

The proposed system uses the FMCW radar EVALKIT SMR-334, manufactured by InnoSenT, 24 GHz. This provides the fast Fourier transform (FFT) result of the bit frequency, representing the difference between the transmitted and received signals as a measurement value. Unlike the conventional method, the proposed scheme does not use distance estimation. We used these FFT measurement values as the input to deep learning pattern matching for estimation of both location and posture of a target.

**Figure 2:**
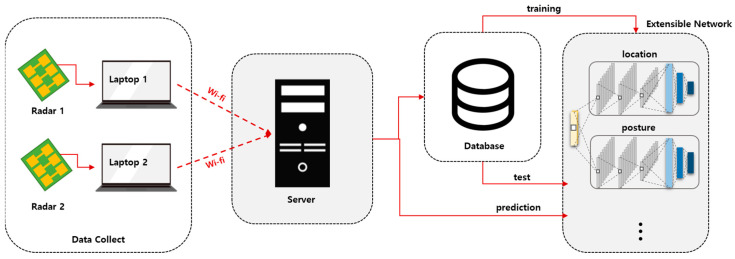
Proposed simultaneous location and posture estimation system.

**Figure 3:**
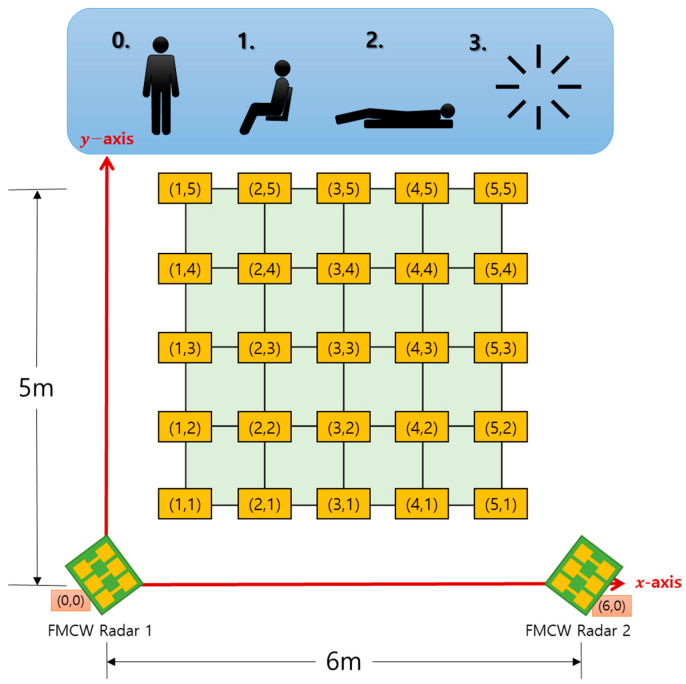
Experimental schematic of the two-dimensional (2D) location and posture estimation.

Figure 2 depicts the developed simultaneous location and posture estimation system, which comprises two FMCW radars and a server. Figure 3 illustrates a schematic of the experimental setup used for evaluating the conventional and proposed schemes. As indicated in the figure, two FMCW radars were placed at both ends of the x-axis, and the received data were collected to estimate the location and posture of the human target, positioned at one of 25 different points over a monitoring area of 5 m × 6 m. The time-series radar data were collected using the developed system for the empty state and three different postures at each location. The collected data were divided into training and testing datasets to evaluate the proposed scheme. The same experimental data were used for both conventional and proposed schemes.

### 3.3. Conventional Scheme

**Figure 4:**
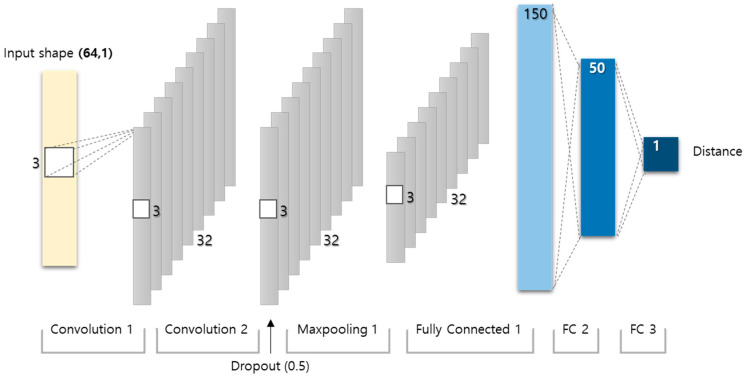
Convolutional neural network (CNN) for distance estimation.

**Figure 5:**
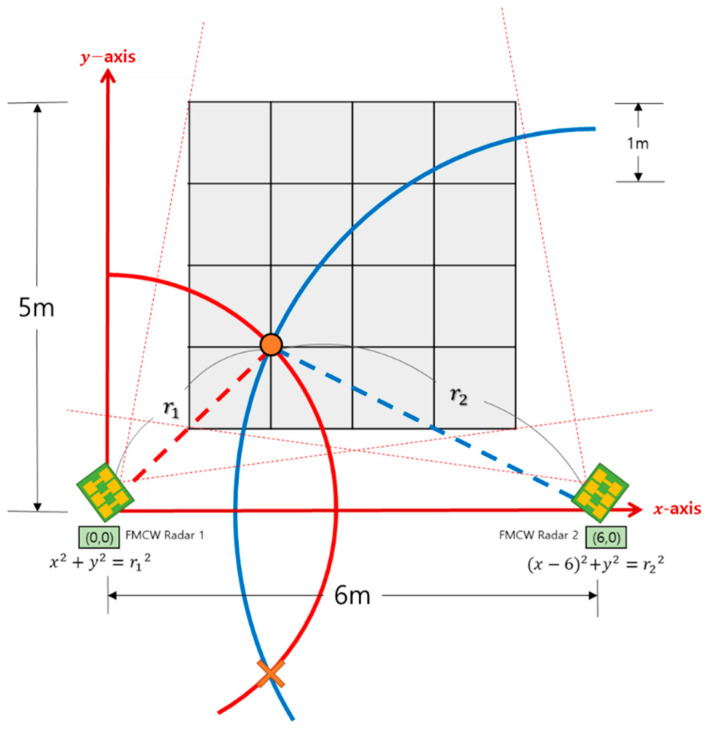
Conventional 2D localization scheme based on the bilateration method.

The conventional multilateration scheme for 2D localization is based on the distance estimation between the radar and target. The distance can be calculated by multiplying the estimated delay time with the propagation speed of the radio wave. As the multilateration method is based on range estimation, its localization performance relies on the accuracy of the estimated distance between the radar and target. To enhance the accuracy of delay time estimation, a conventional DL-based distance estimation scheme was employed for the DFL scheme which uses the FMCW radars [29] (Figure 4). Two circles with different radii (r1 and r2) can be obtained using the estimated distance at the pre-determined position of the radars. Subsequently, the intersection of the two circles was determined to be the location of the target, as depicted in Figure 5.

The posture estimation can be considered as a classifier to distinguish four different categories, namely standing, sitting, lying, and absence. Conversely, the CSP method is considered the conventional device-free posture estimation scheme that exploits FMCW radars. The CSP method is a classic ML scheme that classifies data using linear filters that maximize the variance between classes in the learning data. These linear filters can be obtained via eigenvalue decomposition of the sum of the covariance matrices of the classes. Each linear filter converts the signal to maximize the variance between different classes, which in turn increases the absolute value of the signal component for each axis. Therefore, the CSP method can classify the converted test signal into a class for the axis with the largest component. Apart from the CSP method, a correlation scheme is considered the simplest conventional classifier. It compares the correlation values between the reference and collected signals. Herein, the average signal of the learning data for each class is used as a reference signal, and this scheme classifies the collected signals into classes with the largest correlation values [58]. We also compared the result of using SVM, which is the most popular of the classic machine learning algorithms.

## 4. Proposed Scheme

**Figure 6:**
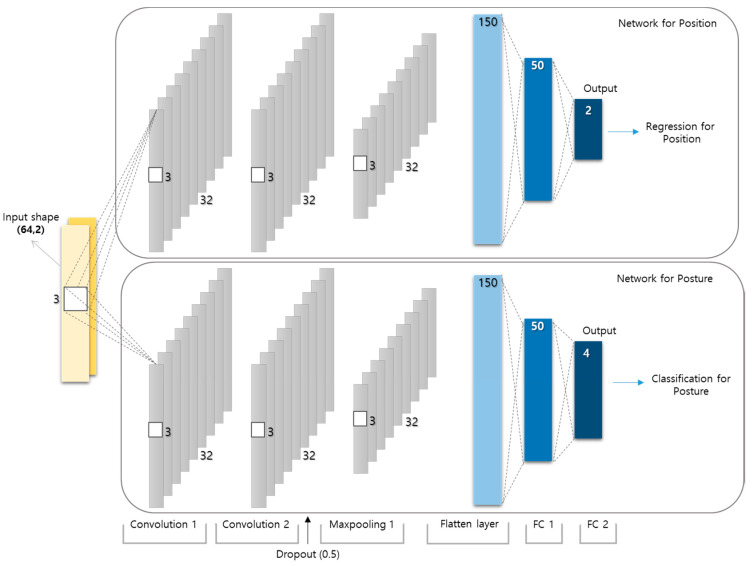
Proposed parallel structure with one-dimensional (1D) CNN.

We propose a DL-based DFL scheme for simultaneous estimation of indoor location and posture using 24-GHz FMCW radars. As the data collected from an FMCW radar are composed of 1D signals, the proposed scheme uses a parallel 1D CNN structure with a regression model for localization and a classification model for simultaneous estimation of the location and posture of a target (Figure 6). Each model does not share layers except the input layer, so it can be learned independently and is designed to be easily extended by using the same network form. As depicted in the figure, the input shape for the CNN model using two radars was set to (64,2) as the data from a single radar are collected in an array of size 64 × 1. The same input data were used for both location and posture estimations in the proposed scheme. Typically, inferring that the features of estimating the location are different from those of estimating the posture is considered reasonable. Therefore, a network of parallel structures is proposed to learn the features separately. A category classifier is known to perform well with cross-entropy as a loss function, whereas the least square error is more adequate as a loss function for estimating the values in successive domains. Therefore, the proposed scheme employs the least square error as a loss function for location estimation, whereas cross-entropy is employed for posture estimation. Table 1 lists the best-performing hyperparameters selected based on previous studies [18,29]. A dataset with 19,124 data points was collected for 25 points and divided into training, validation, and test data in the ratio of 64:20:16. The training and validation data were used to learn the proposed DL model, whereas the test data were used to evaluate the performance of the proposed scheme and were not involved in learning. All data points were randomly divided into four classes because if the data are not split by class, an empty class may occur among the four classes. We combined two simple networks instead of building a complex one to make it easy to expand the system. A basic model used in the system is shown in Figure 6. As depicted in the figure, the model for location estimation and the model for posture estimation are exactly same except the output layer. These models are selected because of their simplicity and lightness. As it mentioned above, each model can be trained separately because they do not share layers nor parameters. In the experiment, we trained the model for localization first by the data labeling with locations, and then trained the model for posture by the same data labeling with postures. These models share the same input so they can work at the same time.

## 5. Performance Evaluation

The proposed system was evaluated experimentally, and the performance of the proposed scheme was compared with that of conventional schemes under identical conditions.

### 5.1. Experimental Environments

**Figure 7:**
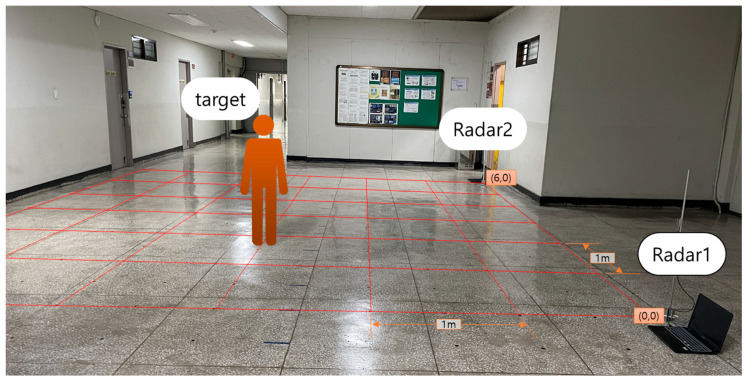
Schematic of the experimental configuration using two frequency-modulated continuous-wave (FMCW) radars.

**Figure 8:**
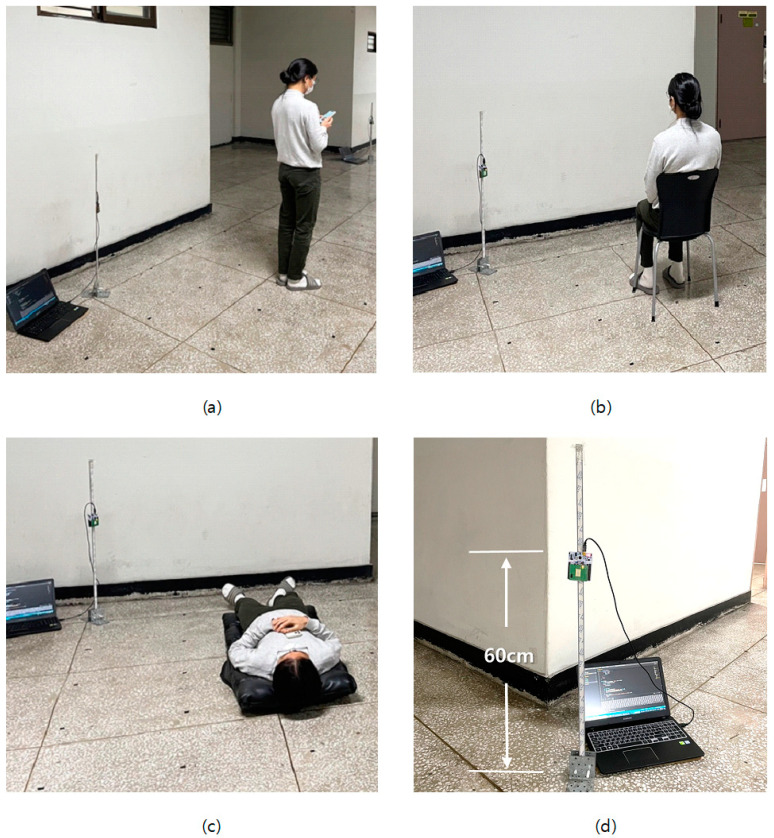
Three different postures of a target, namely (**a**) standing, (**b**) sitting, and (**c**) lying. (**d**) The FMCW radar configuration.

To evaluate the performance of the proposed scheme in actual indoor environments, experiments were performed in a 6.7 m wide, 13.7 m long, and 2.55 m high open corridor on the sixth floor of the general office building at Kwangwoon University. The target in the experiments was a human, and two FMCW radars were placed at each end of the corridor, as depicted in Figure 7. The experiments were conducted by moving the target from point (1,1) to point (5,5), and the data were collected at all 25 points using the two FMCW radars. We performed pre-experiments over 3 months for 30 s, 1 min, 2 min, 3 min, 4 min, and 5 min to decide data collection time. According to the results of experiments, the data were collected for 1 min at each point for three postures, namely standing, sitting, and lying. Furthermore, an additional 100 data points of the empty space were collected in the absence of the target. Figure 8 depicts the three different postures of the target and the FMCW radar configuration fixed at a height of 60 cm from the floor. FFT data from a single radar were collected in an array of size 64 × 1, and the input shape for the proposed CNN model using two radars was set to (64,2).

### 5.2. Experimental Results

**Figure 9:**
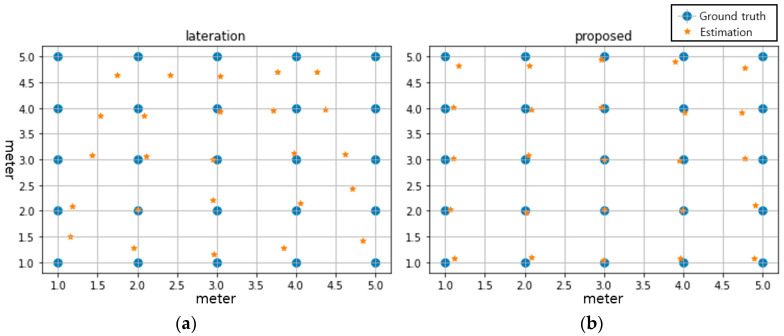
Comparison of localization results obtained from the (**a**) conventional and (**b**) proposed schemes.

Initially, the convergence and accuracy of the parallel 1D CNN model were evaluated. Subsequently, the localization performance of the proposed scheme was investigated. After learning the proposed scheme using the experimental data for different postures at all 25 points, the localization performance was evaluated using a randomly mixed test set. Table 2 summarizes the comparison of the average localization error using the test data of both the conventional and proposed schemes collected at different postures. As indicated in the table, the average localization error of the proposed scheme is approximately 0.23 m, whereas that of the conventional scheme is approximately 0.65 m. This verifies that the proposed scheme outperforms the conventional scheme at all postures. Figure 9 depicts a comparison of the localization results of the conventional and proposed schemes at the same level of signal to noise ratio (SNR). The analysis of the experimental results determined that the variance of the localization error is larger than the sum of the variance of the distance estimation error for each radar. Particularly, in the case of a location outside the field of view (FOV), which is 43 degrees, of the radar, such as, points (1.0, 5.0) and (5.0, 1.0), the difference in accuracy is greater, as shown in the Figure 9a. Therefore, the accuracy of the overall localization decreases in the conventional scheme if the distance estimation error is large even for only one of the two radars. Meanwhile, wireless signals experience reflections, diffractions, and scatterings in complex indoor environment and can spread over a wider area in an indoor propagation environment. As for the considered FMCW radar signal in some places outside of the FOV, we still can receive the radar signal at those reference points even though the signals are not clean. The proposed CNN scheme possesses powerful feature extraction, and it can comprehensively learn features from the input data, which can provide more enhanced performance than conventional methods that need the specification of information such as range information. Note that there is a possibility that an error may be increased if the SNR of received signal is decreased, because the energy of the reflected signal from the target is decreased as the traveled distance increases.

Both the location and posture of the target were estimated simultaneously using the proposed scheme. As the proposed scheme comprises a classification model for posture estimation, four different categories, namely standing, sitting, lying, and absence, were set to be learned. Both FP and FN errors were considered to evaluate the accuracy of the posture estimation. The FP error occurs when a positive value is determined while the true value is negative. Conversely, the FN error occurs when a negative value is determined while the true value is positive. As these two errors typically exhibit a trade-off relationship, they must be compared when evaluating the performance of posture estimation. Table 3 summarizes the comparison of the posture estimation errors for the conventional and proposed schemes. As indicated in the table, the average posture estimation error of the proposed scheme is approximately 1.7%, whereas those of the conventional correlation, CSP and SVM schemes are approximately 54.8%, 42%, and 10%, respectively. We also determined that the absence of the target is detected with higher accuracy than those of the different postures in all schemes. The results validate that the proposed scheme outperforms the conventional scheme in terms of posture estimation as well. Therefore, the proposed DL-based classifier can achieve relatively high accuracy because it learns nonlinear characteristics, whereas conventional schemes are linear classifiers.

## 6. Conclusions

DFL schemes are increasingly employed in various applications, such as security systems, safety monitoring systems, patient or elderly health monitoring systems, search-and-rescue operations, and posture recognition systems in hospitals or disasters. Although camera-based DFL schemes are commonly used for localization and posture estimation of a device-free target, the DFL scheme with radars has recently attracted attention because of its relatively low risk of privacy issues and robustness in dark or smoky environments.

In this study, we propose a DL-based DFL scheme for simultaneous estimation of indoor location and posture of a device-free target using 24-GHz FMCW radars. The proposed system involves a parallel CNN structure with a regression model for localization and a classification model for posture estimation. The developed simultaneous location and posture estimation system comprises two FMCW radars and a server. The experimental results indicate that the performance of the proposed scheme is remarkably enhanced compared to that of conventional schemes in terms of both location and posture estimations. In the case of localization, the average localization error of the proposed scheme is approximately 0.23 m, whereas that of the conventional scheme is approximately 0.65 m. Furthermore, the average posture estimation error of the proposed scheme is approximately 1.7%, whereas that of the conventional correlation, CSP and SVM schemes are approximately 54.8%, 42%, and 10%, respectively.

As the proposed scheme can simultaneously estimate the location and posture of a device-free target, it can be used as a quick-response system in various indoor applications, such as patient or elderly health monitoring systems in hospitals and search-and-rescue operations during disasters. In the future, we intend to develop a simplified simultaneous location and posture estimation scheme using a single FMCW radar. Furthermore, we intend to estimate the locations and postures of multiple targets and various SNR conditions. We also plan to conduct research on learning by generating diverse and large amounts of data using generative adversarial networks.

## Figures and Tables

**Table 1:** Hyperparameters used in the proposed scheme.

Parameter Types	Values
Learning rate	0.0001
Training epoch	1000
Batch size	50
Optimizer	Adam
Loss function	MSE for localization Cross-entropy for posture estimation

**Table 2:** Comparison of localization errors.

Schemes	Standing	Sitting	Lying	Average
Conventional	0.65 m	0.62 m	0.68 m	0.65 m
Proposed	0.22 m	0.24 m	0.23 m	0.23 m

**Table 3:** Comparison of posture estimation errors.

Schemes			Standing	Sitting	Lying	Absence	Average
Conventional	Correlation	FN	48.3%	62.5%	55.3%	66.4%	54.8%
		FP	55.8%	56.4%	83.0%	11.0%	
	CSP	FN	30.1%	54.2%	55.6%	38.6%	42.0%
		FP	53.0%	40.3%	55.6%	12.0%	
	SVM	FN	2.1%	4.1%	18.1%	30.5%	10.0%
		FP	8.0%	19.2%	3.1%	0%	
Proposed		FN	1.3%	1.7%	3.5%	0.2%	1.7%
		FP	1.6%	3.3%	1.1%	0.7%	

## Data Availability

Not applicable.
